# Endovascular Stent Placement for Hemodialysis Arteriovenous Access Stenosis

**DOI:** 10.1155/2015/971202

**Published:** 2015-11-16

**Authors:** Brendon L. Neuen, Richard A. Baer, Frank Grainer, Murty L. Mantha

**Affiliations:** ^1^Department of Renal Medicine, Cairns Hospital, Cairns, QLD 4870, Australia; ^2^School of Medicine and Dentistry, James Cook University, Cairns, QLD 4870, Australia

## Abstract

This study aims to report the outcomes of nitinol and polytetrafluoroethylene covered stent placement to treat hemodialysis arteriovenous access stenosis at a single center over a five-year period. Clinical and radiological information was reviewed retrospectively. Poststent primary and secondary patency rates were determined using Kaplan-Meier analysis. Ten clinical variables were subjected to multivariate Cox regression analysis to determine predictors of patency after stent placement. During the study period 60 stents were deployed in 45 patients, with a mean follow-up of 24.5 months. The clinical and anatomical success rate was 98.3% (59/60). Poststent primary patency rates at 6, 12, and 24 months were 64%, 46%, and 35%, respectively. Poststent secondary patency rates at 6, 12, and 24 months were 95%, 89%, and 85%, respectively. Stent placement for upper arm lesions and in access less than 12 months of age was associated with reduced primary patency (adjusted hazards ratio [HR] 5.1, *p* = 0.0084, and HR 3.5, *p* = 0.0029, resp.). Resistant or recurrent stenosis can be successfully treated by endovascular stent placement with durable long-term patency, although multiple procedures are often required. Stent placement for upper arm lesions and in arteriovenous access less than 12 months of age was associated with increased risk of patency loss.

## 1. Introduction

The provision of hemodialysis for patients with end-stage kidney disease requires a functioning arteriovenous vascular access. This is achieved through surgical creation of an arteriovenous anastomosis using either native vessels (arteriovenous fistulae, AVFs) or prosthetic material (arteriovenous grafts, AVGs). With repeated use, these can become dysfunctional, most commonly due to neointimal hyperplasia resulting in stenosis [1]. Vascular access dysfunction continues to be a major cause of morbidity and mortality and represents a significant proportion of the costs associated with hemodialysis care [2].

Percutaneous transluminal angioplasty is commonly used to treat arteriovenous access stenosis and is established practice in many centers around the world [1]. Despite this, lesions treated with balloon angioplasty are prone to restenosis; at 12 months, approximately half of all AVFs require repeated intervention to maintain patency [3, 4]. Factors such as increased lesion length, upper arm AVFs, and younger AVF age have been shown to be associated with patency loss after balloon angioplasty [5]. Specific locations are also more prone to recurrent stenosis, particularly the juxta-anastomosis in radiocephalic AVFs and cephalic arch in brachiocephalic AVFs [6]. In situations of resistant or recurrent stenosis early after angioplasty, endovascular stent placement should be considered and may prolong access patency [7].

The aim of this study was to report our experience using vascular stents (self-expanding nitinol and polytetrafluoroethylene (PTFE) covered stents) for the treatment of resistant and recurrent stenosis and to determine if there were any predictors of patency after endovascular stent placement.

## 2. Methods

The human research ethics committee for the Cairns Hospital and Hinterland Health Service approved the study (HREC/13/QCH/120-872). Informed patient consent was waived due to the retrospective nature of the study. Hemodialysis vascular access and radiology databases were searched to identify all stent placements for vascular access stenosis and to determine long-term patency outcomes.

### 2.1. Setting and Patients

All patients who underwent stent placement for resistant or recurrent hemodialysis access stenosis (both AVFs and AVGs) between January 2008 and December 2012 at Cairns Hospital were included.

### 2.2. Indications for Stent Placement

Indications for the placement of self-expandable nitinol stents (LifeStent and E-Luminexx; Bard Peripheral Vascular) were (1) recurrent stenosis, defined as restenosis of a previously treated lesion within three months of balloon angioplasty, and (2) resistant stenosis, defined as greater than 30% stenosis of the treated lesion after ultra-high pressure balloon inflation at recommended burst pressure.

Indication for the placement of PTFE covered stents (FLUENCY Plus; Bard Peripheral Vascular, Viabahn; GORE, and Advanta; Atrium) was recurrent stenosis within three months of angioplasty in the cephalic arch or central veins, at the discretion of the operator. The cephalic arch was defined as the proximal five centimeters of the cephalic vein. The central veins were defined as the subclavian and innominate veins and superior vena cava.

Arteriovenous access was investigated for recurrent stenosis on the basis of the following standardized monitoring and surveillance techniques: decreased or absent thrill, difficult cannulation, prolonged bleeding time after dialysis, development of collateral veins, excessive dynamic venous pressures on three consecutive occasions, decreased arterial blood flow (fistula blood flow rate < 500 mL/min; Flow-QC; Transonic Systems, Ithaca, New York) or decreased blood flow by more than 25% of baseline, or abnormal recirculation measurements (>10%) [6, 8]. Radiological confirmation was established following diagnostic contrast fistulography.

### 2.3. Definition of Treatment Areas

**Figure 1:**
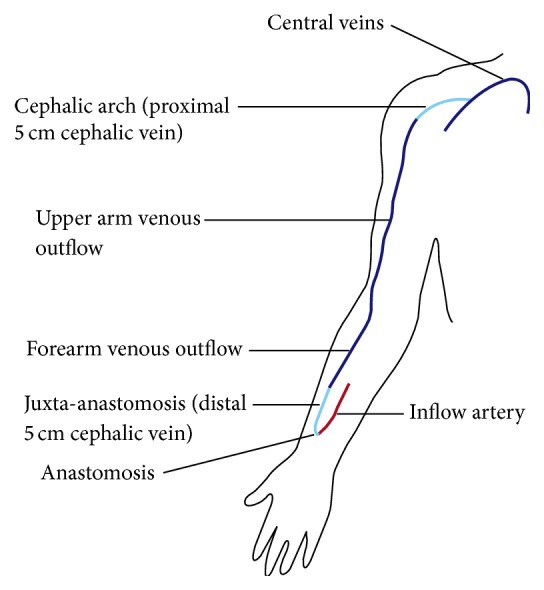
Locations of stent deployment.

Location of stent placement was classified according to a seven-zone system modified from previous studies (Figure 1) [4, 8]: arterial inflow, anastomosis, juxta-anastomosis (distal 5 cm of cephalic vein adjacent to the arteriovenous anastomosis), forearm venous outflow, upper arm venous outflow, cephalic arch (proximal 5 cm of the cephalic vein), and central veins. In the case of brachiobasilic saphenous vein grafts and PTFE grafts, vein-vein anastomotic stenosis and vein-graft anastomotic stenosis were grouped with juxta-anastomotic stenosis.

### 2.4. Procedural Details

Stent placement was performed using standard techniques previously published by our center [6, 8]. Imaging from the cannulation site to the right atrium was obtained using intravenous contrast. A vascular sheath and guide wire were used to obtain access to the lesion. Intermediate, high pressure and ultra-high pressure balloons were used at recommended burst pressure to treat stenotic lesions. Bare metal self-expandable nitinol stents (LifeStent and E-Luminexx; Bard Peripheral Vascular) and covered stents (FLUENCY Plus; Bard Peripheral Vascular, Viabahn; GORE, and Advanta; Atrium) were placed according to the manufacturer's recommended deployment technique. In all instances attempt was made to achieve equal length of the stent on either side of the lesion and postplacement balloon dilatation was performed.

### 2.5. Study Endpoints and Statistical Analysis

Anatomical success was defined as less than 30% residual stenosis after stent placement and demonstrated continuity of the access circuit on contrast study. Clinical success was defined as the ability to provide uninterrupted hemodialysis at prescribed access flow rates for three consecutive sessions using the same access. Procedural complications were recorded and classified according to the Society of Interventional Radiology guidelines [9].

Long-term patency was defined in accordance with the Society of Interventional Radiology reporting standards following treatment with stent placement [10]. Postintervention primary patency was defined as the interval after stent placement until repeated percutaneous intervention is required or the development of access thrombosis. Postintervention secondary patency was defined as the interval after stent placement until the access is surgically declotted, revised, or abandoned. Open surgical repair was employed after failed percutaneous salvage of stenosis, thrombosis, or pseudoaneurysm repair.

Data were recorded and analyzed using SPSS (version 21; IBM, Armonk, New York). Continuous data were reported as mean ± standard deviation. Kaplan-Meier curves were used to determine poststent primary and secondary patency rates. During follow-up, participants were censored because of death with a functioning access, renal transplantation, switch to peritoneal dialysis, or loss to follow-up. Univariate and multivariate Cox regression analysis was performed to determine if there were any predictors of patency after stent placement. Ten variables were analyzed: patient age, gender, indigenous ethnicity, diabetes, history of vascular disease (stroke, coronary or peripheral artery disease), type of stent (self-expandable nitinol or PTFE covered stents), access age (time from creation to stent placement), type (native fistula or native vein/prosthetic graft), lesion location (upper arm or forearm), and antiplatelet therapy.

## 3. Results

During the five-year study period, 60 stent procedures were performed in 45 cases of dysfunctional arteriovenous access in 45 patients. The mean patient age was 57 years and brachiocephalic AVFs were the most common type of access (64%) (Table 1). The majority of patients (60%) were of Aboriginal and/or Torres Strait Islander ethnicity. In 51 cases, the treated lesion had undergone angioplasty on one previous occasion with recurrence of stenosis within three months. In six cases, the treated lesion had been treated with angioplasty on two occasions, although stenosis had recurred beyond three months after the first angioplasty. In three instances a stent was placed during the index procedure due to resistant stenosis despite ultra-high pressure balloon inflation at the recommended burst pressure.

The location of stent placement according to type of arteriovenous access is presented in Table 2. Juxta-anastomotic stenosis was the most commonly treated area in radiocephalic AVFs. In brachiocephalic AVFs, the cephalic arch was the most frequent site of stent deployment.

The anatomical and clinical success rate was 98.3% (59/60 procedures). Complications occurred in four cases. Minor complications included one venous spasm and one access hematoma that did not require specific treatment and extravasation of contrast in another case that settled with balloon tamponade. One procedure was unsuccessful because of failed percutaneous thrombolysis, and stent placement was abandoned. No major complications occurred.

**Figure 2:**
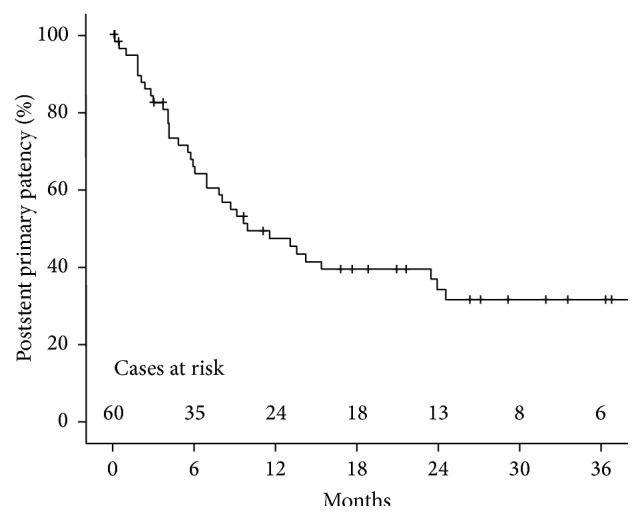
Kaplan-Meier estimate of postintervention primary patency after stent placement.

**Figure 3:**
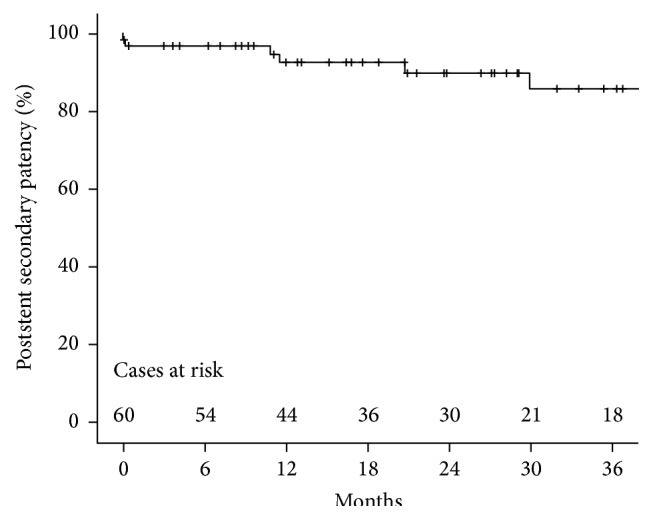
Kaplan-Meier estimate of postintervention secondary patency after stent placement.

Mean follow-up was 24.5 months (range: five days to 4.8 years). One patient was lost to follow-up 30 months after stent placement due to relocation to another hospital. The mean poststent primary patency was 14 months (standard deviation ± 13 months) and the mean poststent secondary patency was 25 months (standard deviation ± 18 months). Poststent primary patency at 6, 12, and 24 months was 64%, 46%, and 35%, respectively (Figure 2). Poststent secondary patency at 6, 12, and 24 months was 95%, 89%, and 85%, respectively (Figure 3).

The mean poststent primary patency for both bare metal and covered stents was 14 months (standard deviation ± 14 and 12 months, resp.). The mean poststent secondary patency for bare metal stents was 29 months (standard deviation ± 20 months). The mean poststent secondary patency for covered stents was 18 months (standard deviation ± 11 months).

**Figure 4:**
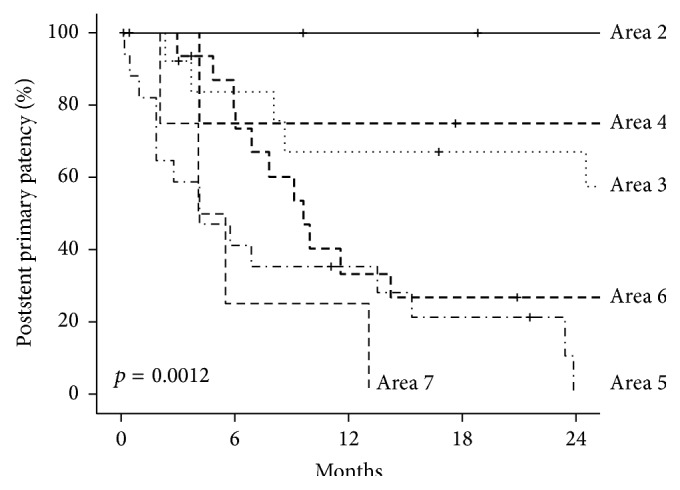
Postintervention primary patency for stents placed at seven treatment areas. Area 2: arteriovenous anastomosis; Area 3: juxta-anastomosis; Area 4: forearm venous outflow; Area 5: upper arm venous outflow; Area 6: cephalic arch; Area 7: central veins.

Poststent primary patency rates for each of the seven treated areas are presented in Figure 4. There was a significant difference in poststent primary patency between the seven treatment areas (log-rank *p* = 0.00012). Stent placement used to treat more central lesions above the elbow, especially the upper arm venous outflow, cephalic arch, and central veins, tended to result in poorer outcomes compared to juxta-anastomotic stenosis and forearm venous outflow lesions.

During the follow-up period, 25 cases (42%) did not require any further interventions to maintain patency. Thirty-five cases (58%) required multiple further interventions; additional 99 interventions were performed to maintain patency, equating to an average of 2.2 further interventions per patient after the index stent deployment. The anatomical and clinical success rate was 98% (97/99 interventions). Twelve cases required one additional intervention to maintain patency. Five cases required further two interventions, seven cases required further three interventions, two cases required further four interventions, three cases required further five interventions, two cases required further six interventions, and two cases required further seven interventions. Fifty-eight out of 99 (59%) of the additional interventions to maintain patency were related to in-stent or peri-stent restenosis.

In multivariate Cox regression, upper arm lesions and access age less than one year at stent insertion were the only factors associated with reduced postintervention primary patency (adjusted hazards ratio [HR] 5.1, 95% confidence interval [CI] 1.5–17, *p* = 0.0084, and HR 3.5, 95% CI 1.5–7.8, *p* = 0.0029, resp., Table 3). There was no significant association between any of the other tested variables and postintervention primary patency (Table 3).

## 4. Discussion

Resistant or recurrent stenosis throughout the access circuit poses challenges in terms of providing optimal hemodialysis treatment. In this study, stent placement to treat upper arm lesions and in access less than 12 months of age was associated with increased risk of primary patency loss. Twelve-month poststent primary and secondary patency rates of 46% and 89%, respectively, were achieved, comparable to studies published in the interventional radiology and vascular surgery literature [1].

Certain anatomical regions of the access circuit are particularly prone to the development of resistant or recurrent stenosis. The vein immediately adjacent to the arteriovenous anastomosis (commonly referred to as juxta-anastomosis) is a common site of stenosis due, in part, to injury sustained at the time of “swinging” the vein to form the anastomosis from the vein's original anatomical location [11]. The proximal portion of the cephalic vein (known as the cephalic arch) is similarly problematic due to the anatomy/morphology of the vein, presence of valves, and extrinsic compression from the clavipectoral fascia, all of which limit vascular remodeling [12]. In this study, more than half of all stents (56%) were at these two discrete locations.

This study found that more central lesions such as those throughout the upper arm venous outflow, cephalic arch, and central veins had poorer patency after treatment with stents compared to juxta-anastomotic lesions (for all types of access) and forearm venous outflow lesions (Figure 4). This reinforces clinical observations that brachiocephalic AVFs can be particularly problematic and prone to recurrent stenosis, particularly at the cephalic arch. This is consistent with one of the largest studies reporting the outcome of 135 stent procedures, which found the mean time to reintervention was significantly longer for forearm AVFs compared to upper arm AVFs [13].

This finding was confirmed after adjustment for other risk factors in multivariate Cox regression, where stent placement in upper arm lesions was associated with reduced poststent primary patency compared to forearm lesions. This may be related to high flow rates and possibly reflect the role of hemodynamic stress in the development of neointimal hyperplasia. Our findings are supported by studies of both balloon angioplasty and stent placement that reported that interventions in upper arm access were associated with reduced postintervention primary patency [8, 14, 15]. However, this association has not been consistently reported [16, 17] and remains to be confirmed in larger prospective studies.

Stent placement in arteriovenous access less than 12 months of age was associated with reduced postintervention primary patency. AVFs that develop stenosis in the first six to 12 months after creation are likely to be intrinsically defective; the early development of stenosis in a previously healthy vein after arteriovenous access formation is a reflection of accelerated neointimal hyperplasia [5]. It is unsurprising then that restenosis is more likely after stent insertion for these patients who developed problematic stenoses in relatively new AVFs.

Although antiplatelet therapy has been shown to reduce access thrombosis [18], we found no association between antiplatelet therapy and poststent patency. Whilst antiplatelet therapy has endothelial protective benefits in the arterial system, similar effects in the venous system have yet to be demonstrated.

The observational nature of this study precluded our ability to definitively answer the question of which type of stent provides superior patency. Covered stents were only used to treat more proximal lesions such as cephalic arch and central stenosis. On the other hand, nitinol stents were used throughout the access circuit. One small randomized trial found that covered stents provided superior patency compared to nitinol stents for the treatment of recurrent cephalic arch stenosis [19]. A recently published randomized trial also found that covered stent placement for cephalic arch stenosis provided both superior access circuit primary patency and target lesion primary patency compared to balloon angioplasty alone [20]. This builds upon increasing evidence, most notably the FLAIR trial [21] and presented results of REVISE and RENOVA, that covered stents prolong access circuit patency in graft-vein anastomotic stenosis compared to balloon angioplasty. The results from this study are likely to reflect the small number of covered stents used and discrepancies in the location of covered stent placement.

This study demonstrated that secondary patency, which reflects continued function of the access, was 85% two years after initial stent placement. In order to provide sustained dialysis using the same access, an average of 2.2 additional interventions per access were required. The vast majority of additional interventions were balloon angioplasties. Of these, approximately 41% were performed for newly developed lesions, slightly less than previously reported in other studies [16]. In most cases, angioplasty was required at the site of stent placement either for in-stent or for peri-stent stenosis.

It is important to note that endothelial injury from repeated balloon angioplasty contributes to the formation of neointimal hyperplasia. Previous studies have demonstrated that repeated angioplasty is associated with increasing cellular proliferation of treated lesions [22]. Aggressive smooth muscle and myofibroblast proliferation due to stent related luminal mismatch causing turbulent flow and altered hemodynamics are important in the pathogenesis of in-stent and peri-stent restenosis [23–25]. The presence of the stent itself also contributes to the pathogenesis of neointimal hyperplasia by attracting macrophages and inducing the expression of a cascade of proinflammatory cytokines [24].

Despite the need for repeated interventions at different intervals, there are several potential advantages of using endovascular stents in comparison to open surgical repair. Stent placement avoids the need for hospitalization; all procedures were performed in a radiology angiographic suite without interruption to patients' dialysis schedules. They are particularly useful for the treatment of central stenosis [26]. Open surgical repair of these lesions is technically complex and highly demanding of patients and is usually only considered once endovascular options are exhausted or ineffective [26]. Demographic changes in dialysis populations mean that more elderly patients with vascular comorbidities are commencing hemodialysis [27]. It is vitally important to preserve sites for future access creation in these patients with relatively poor peripheral vasculature.

There are some important considerations required prior to stent placement. For juxta-anastomotic stenosis in radiocephalic AVFs, surgical revision with formation of a more proximal anastomosis may be preferred because there is an adequate length of cephalic vein available for cannulation in comparison to upper arm AVFs [28]. Juxta-anastomotic stenosis in upper arm access is often located at the elbow flexure crease, which can make stents prone to fractures [1]. Similarly, at the cephalic arch, repeated movement of the upper arm girdle can lead to stent migration, particularly if the stent has not endothelialized, resulting in axillary vein thrombosis and occlusion [1]. Complications such as stent graft infection and damage from cannulation have also been reported [29]. We did not observe any of these serious complications in our study.

This was a retrospective study, with the inherent drawbacks of such an approach. Comparison with other percutaneous interventions (e.g., drug-eluting and cutting balloon angioplasty) and surgical repair were beyond the scope of this study. Without this comparison, it was not possible to extrapolate from the data that secondary patency rates observed were directly attributable to stent placement. It is likely that other confounding factors specific to our center such as a dedicated nurse practitioner vascular access coordinator, access surveillance protocols, and an interventional nephrology led service model influenced the reported outcomes. The cost-benefit of repeated angioplasties compared to surgical correction is an important consideration that warrants further investigation. Future randomized trials are needed to assess the utility of covered stents to treat recurrent stenosis, particularly at the cephalic arch in patients with brachiocephalic AVFs.

## 5. Conclusion

This study demonstrated that resistant or recurrent stenosis could be successfully treated by endovascular stent placement, although multiple procedures are required to maintain patency. Stent placement for upper arm lesions and in arteriovenous access less than 12 months of age was associated with reduced postintervention primary patency.

## Figures and Tables

**Table 1:** Baseline patient characteristics and intervention details.

Patients	45
Male (%)	53
Mean patient age (years)	57 ± 11
Mean follow-up (months)	25 ± 18
Indigenous ethnicity^1^ (%)	60
Access type	
Brachiocephalic AVF	29
Radiocephalic AVF	11
Brachiobasilic saphenous vein graft	4
Brachiobasilic PTFE graft	1
Stent type	
Nitinol stent	44
PTFE covered stent	16
Stent location (%)	
Arterial inflow	0
Anastomosis	5
Juxta-anastomosis	28
Forearm venous outflow	7
Upper arm venous outflow	25
Cephalic arch	28
Central veins	7

^1^Aboriginal and/or Torres Strait Islander ethnicity.

**Table 2:** Stent location according to access type.

Radiocephalic AVFs (*n* = 15)	
Arterial inflow	0
Anastomosis	3
Juxta-anastomosis	7
Forearm venous outflow	4
Upper arm venous outflow	0
Cephalic arch	0
Central veins	1
Brachiocephalic AVFs (*n* = 35)	
Arterial inflow	0
Anastomosis	0
Juxta-anastomosis	7
Upper arm venous outflow	9
Cephalic arch	17
Central veins	2
Brachiobasilic saphenous vein/PTFE grafts (*n* = 10)	
Arterial inflow	0
Anastomosis	0
Vein-vein or vein-graft anastomosis	3
Upper arm venous outflow	6
Central veins	1

**Table 3:** Predictors of postintervention primary patency after stent insertion.

Variables	Unadjusted Cox analysis		Adjusted Cox analysis	
	*p* value	Hazards ratio (95% CI)	*p* value	Hazards ratio (95% CI)
Patient age	0.78	1.0 (0.96–1.0)	—	—
Male gender	0.20	1.6 (0.79–3.0)	—	—
Indigenous ethnicity^1^	0.49	0.79 (0.40–1.5)	—	—
Diabetes	0.62	1.3 (0.52–3.0)	—	—
Vascular disease	0.85	1.1 (0.54–2.1)	—	—
Native AVF	0.11	0.52 (0.24–1.2)	—	—
Access age <12 months	**0.0043**	**3.1 (1.4–6.9)**	**0.0029**	**3.5 (1.5–7.8)**
Upper arm lesion	**0.0067**	**5.2 (1.6–17)**	**0.0084**	**5.1 (1.5–17)**
Covered stent	0.43	0.73 (0.33–1.6)	—	—
Antiplatelet	0.99	1.0 (0.51–1.9)	—	—

^1^Aboriginal or Torres Strait Islander ethnicity.
